# Teeth Erupted into the Antrum.—Inverted Molar

**Published:** 1900-02-15

**Authors:** M. H. Fletcher

**Affiliations:** Cincinnati


### TEETH ERUPTED INTO THE ANTRUM.—INVERTED MOLAR.

BY M. H. FLETCHER, D.D.S., M.D., CINCINNATI.

*Case I.* In my investigation of diseased antrum, I have seen two teeth erupted into the antrum. The first I ever saw was a surgical case in which a cuspid was taken from the left antrum, the patient having suffered for years with pain, finally swelling, great discharge of pus. The surgeon to whom the patient applied, invited me to be present at the operation. A great portion of the superior maxillary bone was removed; a cuspid tooth had been turned back, the distal proximal surface having been exposed in the antrum, had decayed and the pulp had died, and an abscess had formed; there was a great deal of pus in the locality, consequently he suffered a great deal.

There being no way to get at the tooth, no knowledge of the location of tooth, the surgeon was obliged to perform quite an extensive surgical operation, and the patient lost most of the superior maxillary bone.

*Case II.* was one which caused a different kind of trouble; it was brought to my attention by Dr. P. S. Conner, of our city. A third molar had become inverted, the crown having presented itself toward the nose, slightly upward and pressing against the wall upon which the turbinated bones are found. This produced irritation, which resulted in a new growth of bone—this new growth completely filling the antrum. The superior maxillary bone was dissected out and found to be solidified completely, pushing the socket of eye up, and the teeth were pushed down so that the articulation was entirely out of shape.

The history of this case taught me this lesson, that whenever we have obscure pain in this locality, it is well to investigate very thoroughly. One of the most satisfactory means of diagnosing such a condition is that of mouth illumination. We can all now get small mouth lamps which illuminate very well, and in order to make the diagnosis a three candle-power lamp with a little practice will make it is easy to tell if the tissues are normal.

On Saturday of last week I operated on a case in which the patient had suffered with extreme pain in the region of the canine fossa; on examination I found a considerable amount of spongy bone underneath the soft tissues; the illumination showed a shadow in this place. The illumination will throw a shadow or different light through abnormal tissue. I have no doubt that if it had been used in the cases above referred to better diagnosis could have been made.

The suggestion I would make in handling cases of this kind is get a lamp illumination.

